# VESNA: an open-source tool for automated 3D vessel segmentation and network analysis

**DOI:** 10.1186/s12859-025-06270-6

**Published:** 2025-10-21

**Authors:** Magdalena Schüttler, Leyla Doğan, Jana Kirchner, Süleyman Ergün, Philipp Wörsdörfer, Sabine C. Fischer

**Affiliations:** 1 Faculty of Biology, Center for Computational and Theoretical Biology, Julius-Maximilians-Universität Würzburg, Klara-Oppenheimer-Weg 32, 97074 Würzburg, Germany; 2 Faculty of Medicine, Institute for Anatomy and Cell Biology, Julius-Maximilians-Universität Würzburg, Koellikerstr. 6, 97070 Würzburg, Germany; 3 Istanbul Atlas University, Istanbul, Turkey

**Keywords:** Image processing, Image analysis, Fiji, ImageJ, Blood vessel, Angiogenesis, Organoids, Hydrogel culture, 3D tissue culture, Fluorescence imaging

## Abstract

****Background**:**

Vasculature is an essential part of all tissues and organs and is involved in a wide range of different diseases. However, available software for blood vessel image analysis is often limited: Some only process two-dimensional data, others lack batch processing, putting a time burden on the user, while still others require tightly defined culturing methods and experimental conditions. This highlights the need for software that has the ability to batch process three-dimensional image data and requires few and simple experimental preparation steps.

****Results**:**

We present VESNA, a Fiji (ImageJ) macro for automated segmentation and skeletonization of three-dimensional fluorescence images, enabling quantitative vascular network analysis. It requires only basic experimental preparation, making it highly adaptable to a wide range of possible applications across experimental goals and different tissue culturing methods. The macro’s potential is demonstrated on a range of different image data sets, from organoids with varying sizes, network complexities, and growth conditions, to expanding to other 3D tissue culturing methods, with an example of hydrogel-based cultures.

****Conclusions**:**

With its ability to process large amounts of 3D image data and its flexibility across experimental conditions, VESNA fulfills previously unmet needs in image processing of vascular structures and can be a valuable tool for a variety of experimental setups around three-dimensional vasculature, such as drug screening, research in tissue development and disease mechanisms.

**Supplementary Information:**

The online version contains supplementary material available at 10.1186/s12859-025-06270-6.

**Fig. 1:**
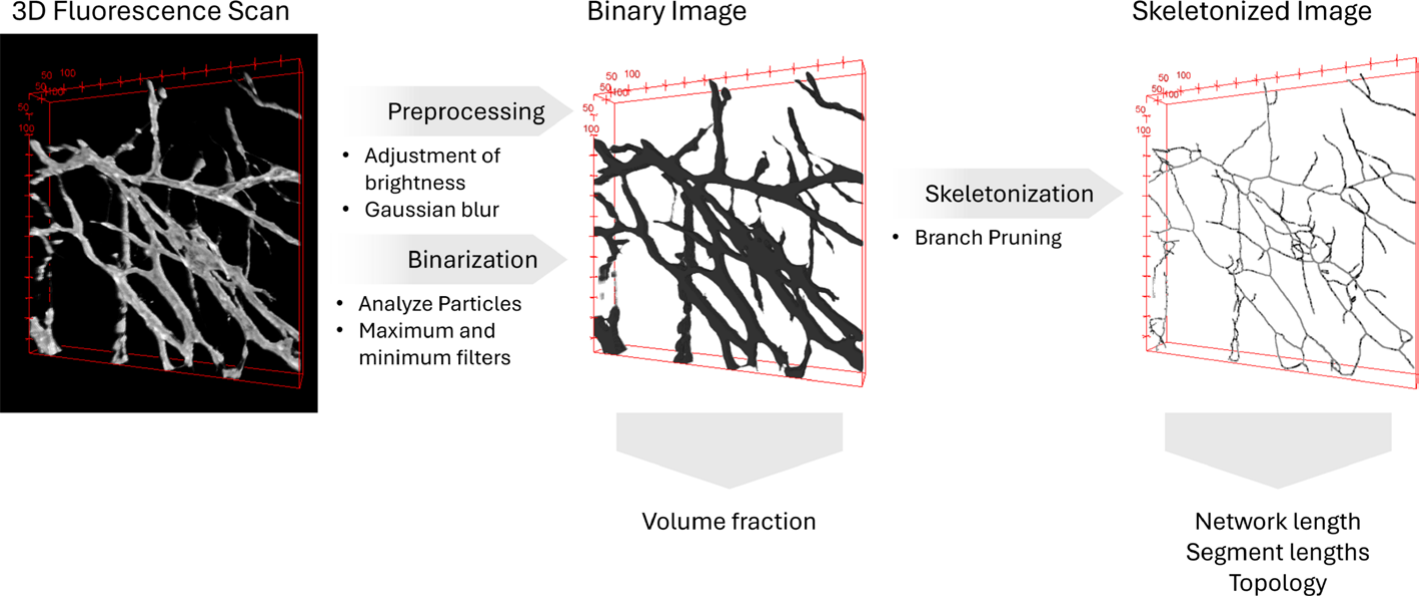
Schematic overview of the three main steps of VESNA’s image processing workflow, the associated postprocessing methods, and the measurements carried out by the macro. Depicted are the raw, binarized, and skeletonized images of one image of Dataset D. The skeletonized image was dilated for better visibility. The red scales specify the image dimensions in $\upmu$m

## Introduction

In recent years, three-dimensional tissue culture techniques, such as organoids, have emerged as powerful tools for studying organ development, function, and diseases. Blood vessel organoids are developed by self-organization, like vasculogenesis. Their organization and branching pattern mirror the tissue vascularization during embryonic development. Beyond its significance in cardiovascular diseases, the vascular system is central to conditions like diabetes [1] and tumor progression [2, 3]. Recent efforts have extended to exploring assembloids and vascularized organoids, enabling the creation of older, larger, and more complex models [3–6].

While significant numbers of organoids can be produced and imaged with relative ease, the manual image processing of the resulting data is often a limiting factor [7, 8].

Early open-source plugins and software tools, such as *AngioQuant* [9], *VESGEN 2D* [10], and *AngioTool* [11], provide semi-automated and automated analyses of two-dimensional images of different imaging modalities. Over time, newer tools like *Quantification* [12], *REAVER* [13], *Angiogenesis Analyzer* [14], and *Q-VAT* [15] have introduced advanced functionalities, focusing mostly on fluorescence microscopy images. *SproutAngio* [16] and *VesselExpress* [17] further include the ability to process three-dimensional images, a feature that is essential for organoid analyses.

Nevertheless, these software tools exhibit notable limitations when applied to blood vessel organoids. For instance, *SproutAngio* lacks the capacity for batch processing, making image processing and analyses time-consuming. Specialized tools like *VesselExpress* or *Automated Sprout Analysis* are optimized for narrowly defined experimental setups such as hydrogel-filled vessels, which are challenging to achieve in organoids. They may also demand additional staining protocols, such as F-actin and nucleus stains, which may not align with the experimental goal, or require imaging modalities that are difficult to apply to organoids, such as bright field microscopy.

These limitations highlight a clear gap, which we address with our macro VESNA (Vessel Segmentation and Network Analysis). Designed explicitly for three-dimensional fluorescence images of vascular networks, VESNA requires only a vasculature-specific stain, such as CD31. Our macro emphasizes batch processing and minimal user intervention, enhancing efficiency and reducing processing time. VESNA employs a skeleton-based analysis approach, which allows access to the topology of the network structure. This is common for analyzing vascular systems; all previously mentioned tools also use a skeleton-based workflow.

VESNA is free, thus accessible to a large audience, and open-source, making every step of the pipeline transparent and adaptable. To ensure cross-platform functionality and ease of use, it is developed for the open-source platform Fiji (ImageJ) [18]. This allows VESNA to reach the user base already familiar with Fiji and enables integration with other Fiji macros and plugins.

We validated VESNA’s performance against ground truth data and evaluated its sensitivity to parameter settings. Furthermore, we demonstrated its ability to process diverse datasets, including drug-treated organoids, organoids cultured under varying growth conditions, and hydrogel-based models. Regardless of image size, network complexity, or organoid age, the macro effectively analyzed vascular network size and structure. This versatility underscores its value in diverse experimental setups, addressing a critical need in vascular organoid research.

## Materials and methods

### Image processing and data analysis

The development of our macro VESNA builds upon the foundational work of *Quantification* by Rust et al. [12]. *Quantification* is a macro for the image processing software Fiji (ImageJ) [18], that allows for automated segmentation of two-dimensional fluorescence microscopy images of vascular networks. VESNA expands on this by enabling the processing of three-dimensional image data that better reflects the complexity of vascular structures *in vitro*, thereby ensuring a more comprehensive representation of biological systems and processes. We included several pre- and post-processing steps to yield high-quality image and measurement data, and introduced batch processing to further automate the image processing workflow, minimizing user involvement during the processing and improving efficiency.

The macro’s image processing workflow can generally be divided into three steps: First, the raw fluorescence microscopy image is preprocessed to ensure the fidelity of later steps. The image is then binarized to define the areas of the vascular structures. Finally, skeletonization simplifies the binary image for measurement (Fig. 1).

#### Preprocessing

During preprocessing, several operations are applied to the raw image to reduce noise and achieve a high quality of the following binarization. First, the image’s brightness is adjusted to ensure the detection of vessels with low fluorescence intensity. A three-dimensional Gaussian blur is utilized to compensate for the highly heterogeneous fluorescence within the vessels in the available images. This ensures the recognition of the vessels as a whole instead of a falsely fragmented segmentation. Both steps can be adjusted using user-defined parameters in the macro interface. The default values of these and all other parameters are listed in Table S1.

#### Binarization

**Fig. 2:**
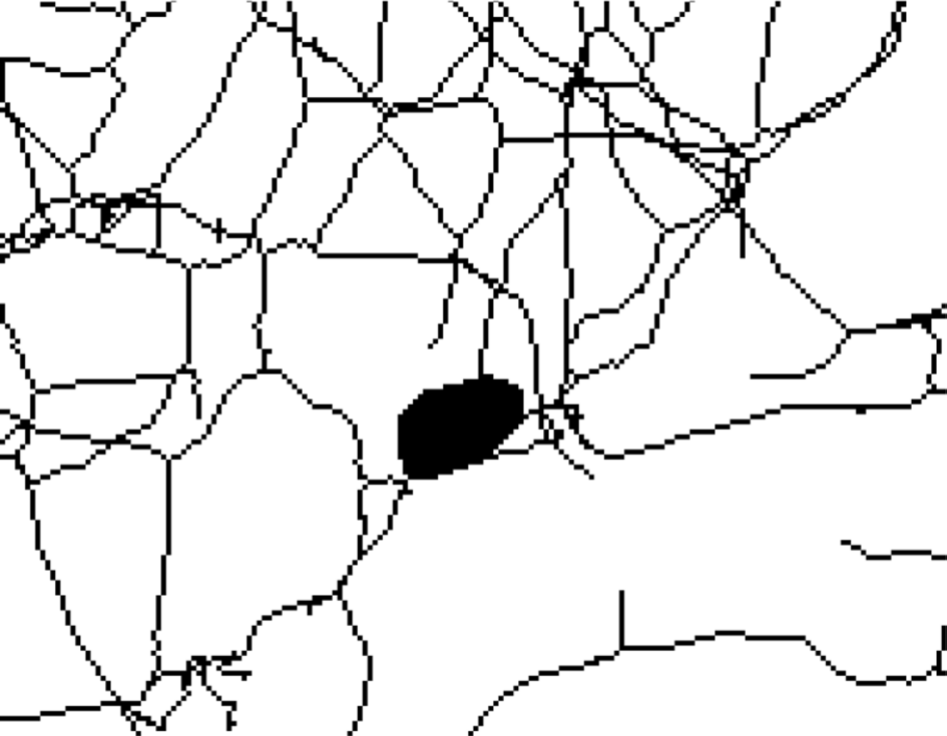
Exemplary cropped Z maximum projection of a skeleton with an artifact that would be removed by the Fill Holes function. The skeleton was generated from Dataset A by omitting the Fill Holes function

Following the preprocessing steps, the vessel structures are segmented. For this, the Yen threshold [19] is used. To improve the fidelity of the resulting binary image, the macro first removes artifacts smaller than a defined pixel value using the Analyze Particles function before applying three-dimensional maximum and minimum filters to connect fragmented vessel structures. Lastly, internal holes in the binary image are removed by the Fill Holes function included in the package *MorphoLibJ* [20]. This step is essential to prevent artifacts in the skeleton that would otherwise be hard to remove (see Fig. 2 for an example).

The resulting binary image is used to calculate the volume fraction of the acquired capillary network. All other measurements are performed based on the skeletonized image.

A machine learning-based binarization via *Trainable Weka Segmentation* [21] was tested as an alternative to the binarization using preinstalled thresholding methods, but was found to require too much processing power at original image dimensions. Scaling of the images led to poor segmentation quality. For this reason, less intensive automated binarization by Yen thresholding was selected to ensure high-quality results while limiting the required processing power and time.

#### Skeletonization

The binary image is then skeletonized. Skeletonization of binary images is a commonly used method in image processing. It simplifies the segmented object to access critical information about its topology and shape. This produces a network of one voxel thick segments connected by junction points and bounded by end points. The plugin *Skeletonize3D* [22] is utilized for this. Small artifactual network segments are removed by a version of the BeanShell script *Prune Skeleton Ends* [23], that we adapted for compatibility with our macro while maintaining its functionality.

Finally, the remaining measurements are performed. The length of the entire vessel network is measured in voxels and voxels per mm³ using the plugin *Analyze Regions 3D*, which is part of the *MorphoLibJ* package [20]. The plugin *AnalyzeSkeleton* [24] allows access to several measurements, including the number of skeletons, segments, junctions, and end points, as well as the segment length.

**Fig. 3:**
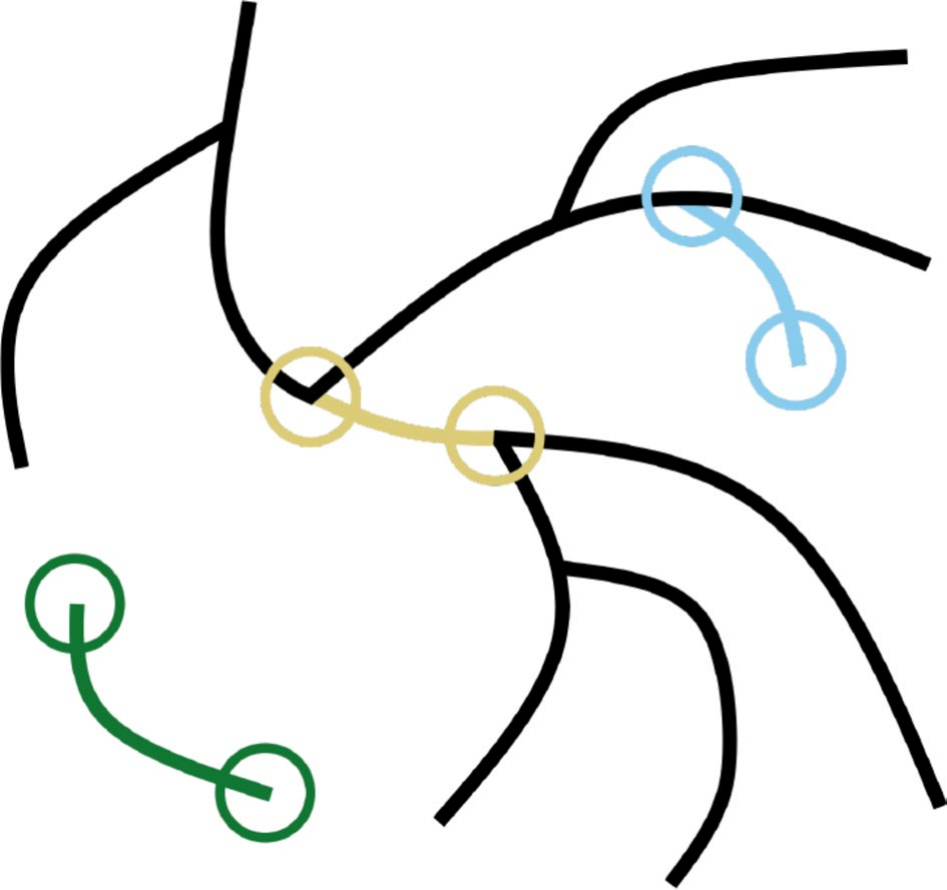
Illustration for explanation of the terms “skeleton” and “segment”. Exemplary segments are highlighted in colors. Circles mark junctions and end points. A skeleton is a group of segments that are directly connected. Hence, the illustration contains two skeletons

In this context, a segment is defined as a part of a skeleton that ends in two junction points, two end points, or one junction point and one end point (Fig. 3). A skeleton is a group of segments that are directly connected. According to this definition, the skeletonized image of one vascular network, for example of one blood vessel organoid, could—and often does—consist of several skeletons. However, the term skeleton is frequently used inconsistently for the entire skeletonized image. To avoid confusion, we will only use the term skeleton to refer to the former definition.

#### Parameter optimization

Image processing with VESNA is fully automated. The only manual step is setting the parameter values for the individual steps of the pipeline. This allows adaptation of the pipeline to the image properties at hand. As a baseline, we have selected default parameter settings that yield decent segmentation quality for most image data presented here. In addition, we established an optimized parameter value set for each data set in this work based on manual comparison between the raw, binary, and skeletonized images. Default and optimized parameter settings for each dataset are listed in Table S1. In this section, we provide a guide for each parameter of our macro, explaining how to identify correct or incorrect values based on VESNA’s returned binary and skeletonized images.

Proper setting of the brightness parameters was verified by visual comparison of the binary image with the raw image. Ideally, the entire vessel structure should be recognized during segmentation without loss of definition in small and detailed structures. Recognition of weakly fluorescing vessels can be improved by lowering the brightness maximum. Conversely, the brightness minimum and maximum can be increased to improve definition. In practice, particularly in images exhibiting nonhomogeneous fluorescence and high background fluorescence, achieving a balance is often challenging. In such cases, implementation of the Subtract Background function can lead to better results. Usually, ensuring accurate segmentation of vessels with high fluorescence should be the priority. Although this can lead to incomplete recognition of weakly fluorescing vessels, this approach will still yield accurate network measurement data for the recognizable regions. In any case, the priority should be based on the specific goals of the experiment.

**Fig. 4:**
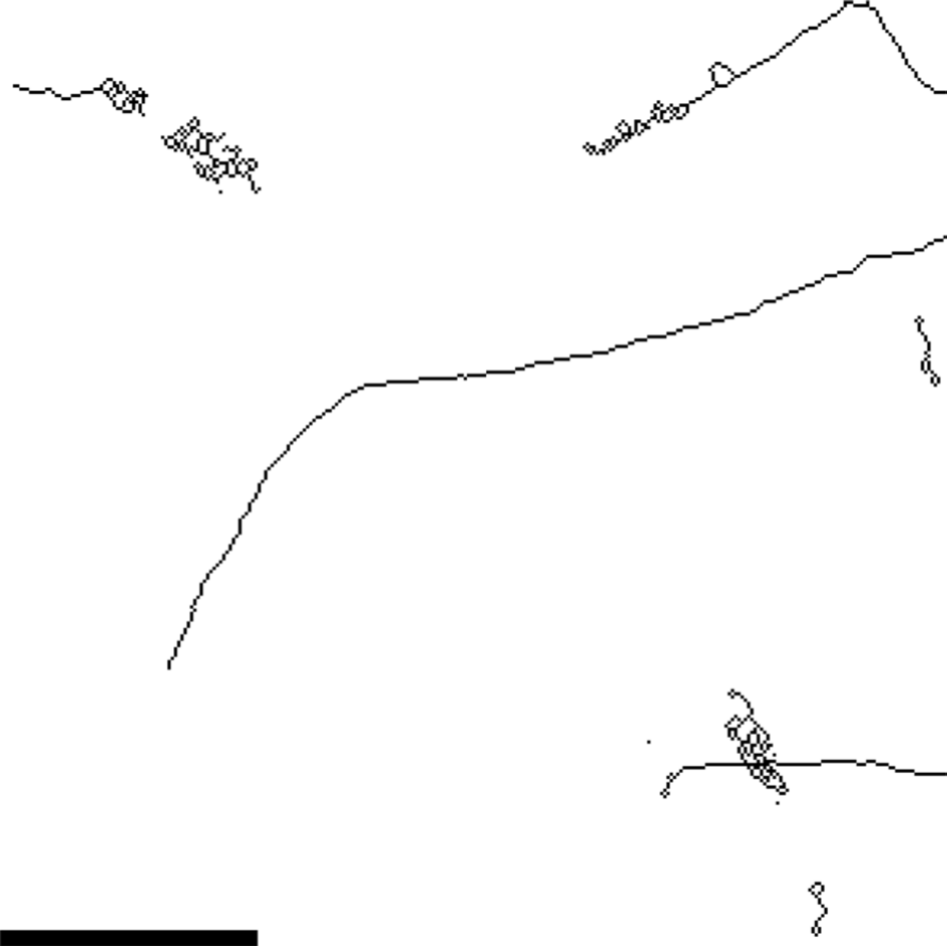
Exemplary image showing artifacts resulting from incorrectly set $\sigma$ parameter for Gaussian blur. Depicted is a cropped Z maximum projection of a skeletonized image of Dataset D that was generated by omitting the Gaussian blur. Scale 50 $\upmu$m

The primary purpose of the maximum and minimum filters, as well as the Gaussian blur, is connecting fragmented vessels in the skeletonized image. Additionally, the Gaussian blur removes small artifactual segments that occur in regions with irregularly shaped vessel structures in the binary image (Fig. 4). Therefore, the setting of these parameters was assessed by comparing the skeleton to the raw image. The parameter values were chosen so that such artifacts or fragmented vessels were minimal, while the detail of the network was maintained.

Verification of the pixel threshold value for the Analyze Particles function was achieved by checking for minor artifacts in the binary or skeletonized image that are not directly connected to larger structures and do not accurately reflect the vascular network observed in the raw image.

Branch Pruning removes any remaining short artifactual segments directly connected to the network structure. The length threshold was considered correct if the skeleton did not show such short artifacts.

#### Computational resources

All analyses presented in this study were conducted on standard low- to mid-range personal computers. VESNA’s most common computational limitation is memory usage, particularly when processing large images, complex networks, or large batch sizes. On a system with 16 GB of RAM, all data could be processed within a few minutes per image. To provide a general estimate of performance, observed processing times for representative images of varying sizes are summarized in Table S2. VESNA uses minimal disk space and does not present CPU-based performance constraints. Based on these observations, we expect VESNA to perform well using standard computers without specialized hardware requirements.

### Generation of sample data

To assess the functionality of VESNA on different types of data, vascular networks were generated in blood vessel organoids and hydrogel-based tissue cultures of varying cell count and age. Additionally, treatment with antiangiogenic substances was performed to demonstrate the possible usage of VESNA in drug screening assays. Dimensions of all processed images are listed in Table S2.

#### Human iPSC culture

Human induced pluripotent stem cells (hiPSCs) were generated from commercially available normal human dermal fibroblasts (Promocell, Heidelberg, Germany) by reprogramming using the hSTEMCCA-lentiviral construct [25, 26]. The hiPSCs are cultured on human embryonic stem cell (hESC)-qualified Matrigel (Corning, New York, NY, USA)-coated culture plates in StemMACS iPS Brew medium (Miltenyi Biotec, Bergisch Gladbach, Germany). The culturing medium is replaced daily. For passaging, cells are dissociated at 80% confluency with StemPro Accutase (Thermo Fisher Scientific, Waltham, MA, USA) for 5 min at 37 $^{\circ }$C to obtain a single-cell suspension. Subsequently, hiPSCs are replated in StemMACS medium, supplemented with 10 nM thiazovivin (TZ) (LC Labs, Woburn, USA).

#### Blood vessel organoid generation

Blood vessel organoids are self-organizing 3D tissue cultures derived from hiPSCs. They consist of a loose mesenchymal connective tissue harboring a hierarchically organized and branching endothelial network. Different approaches were used to generate blood vessel organoids for this study. For Dataset A, blood vessel organoids were generated following a previously established protocol [27]. In brief, hiPSCs are detached from the cell culture dish using StemPro Accutase for 5 min at 37 $^{\circ }$C to obtain a single-cell suspension. Subsequently, 4000 cells per 100 $\upmu$l StemMACS iPS Brew medium (Miltenyi Biotec, Bergisch Gladbach, Germany) supplemented with 10 mM TZ are prepared and pipetted into each well of an agarose-coated 96-well plate. Cells are cultured for 24 h at 37 $^{\circ }$C and 5% CO_2_ in a humidified incubator to allow hiPSC aggregate formation. After 24 h, the medium is changed to mesodermal induction medium (MIM) (Advanced DMEM/F12 (Gibco/Life Technologies, Westham, MO, USA) 100%, L-Glutamine (Gibco/Life Technologies, Westham, MO, USA) 0.2 mM, Ascorbic acid (Sigma-Aldrich, St. Louis, MO, USA) 60 $\upmu$g $\upmu \text {L}^{-1}$, CHIR 99021 (Sigma-Aldrich, St. Louis, MO, USA) 10 $\upmu$m, BMP4 (PeproTech, Cranbury, NJ, USA) 25 ng $\text {mL}^{-1}$) for 72 h. After that, the medium is discarded, and organoids are cultivated in vascular growth medium (VGM) (Neurobasal medium/DMEM-F12 (Gibco/Life Technologies, Westham, MO, USA) (50%/50%), B27 without Vitamin A (Gibco Life Technologies, Westham, MO, USA) 1×, N2-Supplement (Gibco Life Technologies, Westham, MO, USA) 1×, L-Glutamine (Gibco Life Technologies, Westham, MO, USA) 2 mM, Ascorbic acid 60 $\upmu$g $\upmu \text {L}^{-1}$, vascular endothelial growth factor (VEGF) (ProteinTech, Rosemont, IL, USA) 100 $\upmu$g $\upmu \text {L}^{-1}$). After 48 h, the medium is changed to organoid maintenance medium (OMM) (Neurobasal medium/DMEM-F12 (50%/50%), B27 without Vitamin A 1×, N2-Supplement 1×, L-Glutamine 2 mM, Ascorbic acid 60 $\upmu$g $\upmu \text {L}^{-1}$) and organoids are cultured at 37 $^{\circ }$C and 5% CO_2_ in a humidified incubator on a cell culture rocking table. The medium is changed every other day. Vascular networks were analyzed at day 14.

The same protocol was used for Datasets B and C, except that hiPSCs are seeded into custom-made aggrewell-like agarose micromolds instead of agarose-coated 96-wells. To prepare agarose micromolds, 2% agarose (Biozym, Hessisch Oldendorf, Germany) was boiled in water and hot agarose solution was poured into the well of a 12-well culture dish containing a 3D-printed negative master mold. Two different types of negative master molds were used (either with 159 micropillars to create small agarose molds (159 x 700 µm diameter) or with 31 micropillars to create larger agarose molds (31 x 2 mm diameter)). After one hour, the negative master mold was removed from the solidified agarose and the obtained micromold was placed into a well of a 12-well plate (small molds) or 6-well plate (large molds). ${2}-{10} \times 10^{4}$ cells are seeded per small micromold and ${1}-{2} \times 10^{5}$ cells for large molds and cultured for 24 h in StemMACS iPS Brew medium (Miltenyi Biotec, Bergisch Gladbach, Germany) at 37 $^{\circ }$C and 5% CO_2_ in a humidified incubator. 159 iPSC aggregates form in small molds and 31 iPSC aggregates form in large molds. After 24 h, the medium was changed to MIM (Advanced DMEM-F12 (Gibco/Life Technologies, Westham, MO, USA) 100%, L-Glutamine (Gibco/Life Technologies, Westham, MO, USA) 2 mM, Ascorbic acid (Sigma-Aldrich, St. Louis, MO, USA) 60 $\upmu$g $\upmu \text {L}^{-1}$, CHIR 99021 (Sigma-Aldrich, St. Louis, MO, USA) 10 $\upmu$m, BMP4 (Pepro Tech, Cranbury, NJ, USA) 25 ng $\text {mL}^{-1}$) for 72 h. After that, the organoids were removed from the micromolds and transferred into a well of a 6-well plate. The organoids were then cultured in VGM (Neurobasal medium/DMEM-F12 (Gibco/Life Technologies, Westham, MO, USA) (50%/50%), 5% heat-treated fetal calf serum (FCS), B27 without Vitamin A (Gibco Life Technologies, Westham, MO, USA) 1×, N2-Supplement (Gibco Life Technologies, Westham, MO, USA) 1×, L-Glutamine (Gibco Life Technologies, Westham, MO, USA) 2 mM, Ascorbic acid 60 $\upmu$g $\upmu \text {L}^{-1}$, VEGF (ProteinTech, Rosemont, IL, USA) 50 $\upmu$g $\upmu \text {L}^{-1}$) on a cell-culture rocker to avoid aggregation.

For antiangiogenic drug treatment, organoids from small molds were separated into two comparable groups at culture day 8. Subsequently, one group is treated for 72 h with 15 nM sorafenib (Gibco, Karlsruhe, Germany). For the control group, sorafenib is omitted. Analyses of vascular networks were performed after 72 h of sorafenib treatment (culture day 11).

For Dataset B, organoids from small and large molds were compared at culture day 8.

To visualize vessel networks, whole-mount immunofluorescence staining and ethyl cinnamate-based tissue clearing were performed as previously described [28]. A primary antibody directed against CD31 (PECAM1) (Agilent, Santa Clara, CA, USA, mouse, M0823, 1:200) was used to detect endothelial cells. Cy3-conjugated secondary antibodies (Dianova, Hamburg, Germany) were used to visualize the primary antibody. Imaging was performed using the Nikon Eclipse Ti confocal laser scanning microscope (Nikon, Tokyo, Japan) with a long working distance air objective (20×) for taking z-stack images. Nikon NIS Elements Confocal software version 4.13.05 (Nikon, Tokyo, Japan) was used for imaging.

#### Generation of bioprinted hydrogel-based vascular cultures

HiPSCs-derived mesodermal progenitor cells (MPCs) can spontaneously form a loose mesenchymal connective tissue harboring a branching endothelial network after extrusion-based bioprinting using Matrigel as a bioink, as previously reported [29].

For Dataset D, hiPSCs were first converted to MPCs using an adherent 2D cell culture protocol [30]. Therefore, $3 \times 10^{5}$ hiPSCs/$\text {cm}^{2}$ were seeded per Matrigel-coated well of a 6-well plate and cultured for 24 h in StemMACS iPS Brew medium supplemented with 10 mM TZ. Afterwards, the cells were cultured for 3 days in MIM (Advanced DMEM/F12 100%, L-Glutamine 0.2 mM, Ascorbic acid 60 $\upmu$g $\upmu \text {L}^{-1}$, CHIR 99021 10 mM, BMP4 25 ng $\text {mL}^{-1}$) at 37 $^{\circ }$C and 5% CO_2_.

At day 4, cells were dissociated using Accutase. $6 \times 10^{6}$cells were mixed with cold Matrigel solution (3.6 mg $\text {mL}^{-1}$ final protein content in Advanced DMEM/F12) and a homogeneous gel-cell mixture (bioink) was prepared. The mixture was extruded into silicon molds through a 22 G $\frac{1}{4}$ inch lock tip nozzle under a continuous pressure of 10 kPa, using the VIEWEG GmbH brand DC 200 model analog dispenser to form gel disks with a volume of 100 $\upmu$l. The printed disks were kept at room temperature for 45 min. Subsequently, cell-loaded gel disks were washed with PBS and transferred into a cell culture flask containing vascular differentiation medium (Advanced DMEM/F12 100%, 5% heat-treated FCS, 1% Penicillin/Streptomycin (P/S), TZ 10 mM, L-Glutamine 0.2 mM, Ascorbic acid 60 $\upmu$g $\upmu \text {L}^{-1}$, hVEGF-A 60 $\upmu$g $\upmu \text {L}^{-1}$). For whole-mount immunofluorescence analyses, the disks were removed from the culture at days 7 and 17 and washed three times with PBS for 5 min. The disks were fixed with 4% paraformaldehyde (PFA) in PBS for 24 h at 4 $^{\circ }$C and washed again three times with PBS for 30 min to remove residual PFA. After washing, the disks are incubated in blocking buffer (PBS, 4% normal goat serum (NGS, G9023, Sigma-Aldrich, USA), 0.1% Triton X-100) for 3 h at 4 $^{\circ }$C. The samples are incubated with a primary antibody directed against CD31 (PECAM1) overnight at 4 $^{\circ }$C. Afterwards, samples were rinsed three times with PBS and exposed to Cy3-conjugated secondary antibodies to visualize the primary antibody (Dianova, Hamburg, Germany). Imaging was performed using the Nikon Eclipse Ti confocal laser scanning microscope (Nikon, Tokyo, Japan) with a long working distance air objective (20×) for taking z-stack images. Nikon NIS Elements Confocal software version 4.13.05 (Nikon, Tokyo, Japan) was used for imaging.

### Statistical analysis

We used the two-sided Mann–Whitney U test for hypothesis testing with a significance level of $\alpha ^* = 0.05$ and Bonferroni correction. The Bonferroni-corrected significance levels $\alpha$ are listed in Table 1. In the case of the Sensitivity of Parameters (section “Sensitivity of network measurements”), the Branch Pruning parameter does not affect the volume fraction and is thus omitted from the test in this measurement, leading to a separate $\alpha$ value.

The corresponding effect sizes are tested using Glass’s $\Delta$. It is calculated with $\Delta = \displaystyle \frac{|\bar{x}_1 - \bar{x}_2|}{\sigma _2}$, where $\bar{x}_1$ and $\bar{x}_2$ are the mean values of the compared groups 1 and 2, and $\sigma _2$ is the standard deviation of the untreated or control group 2. The resulting Glass’s $\Delta$ values give insight into the extent of the deviation. Reflecting Cohen [31], a value of $\Delta \le 0.2$ indicates a low effect size, $0.2 < \Delta \le 0.5$ a medium effect size, and $\Delta> 0.5$ a high effect size. The statistical analyses were implemented in Python Version 3.11.7.

**Table 1:** Bonferroni-corrected significance levels $\alpha$ calculated for all statistical tests performed

Section	Measurements	$\pmb {\alpha }$
Sensitivity of network Measurements (section “Sensitivity of network measurements”)	Volume fraction	0.0029
	Other	0.0036
Application to sample data (section “Application to sample data”)	All	0.050

## Results

### Image segmentation quality

**Fig. 5:**
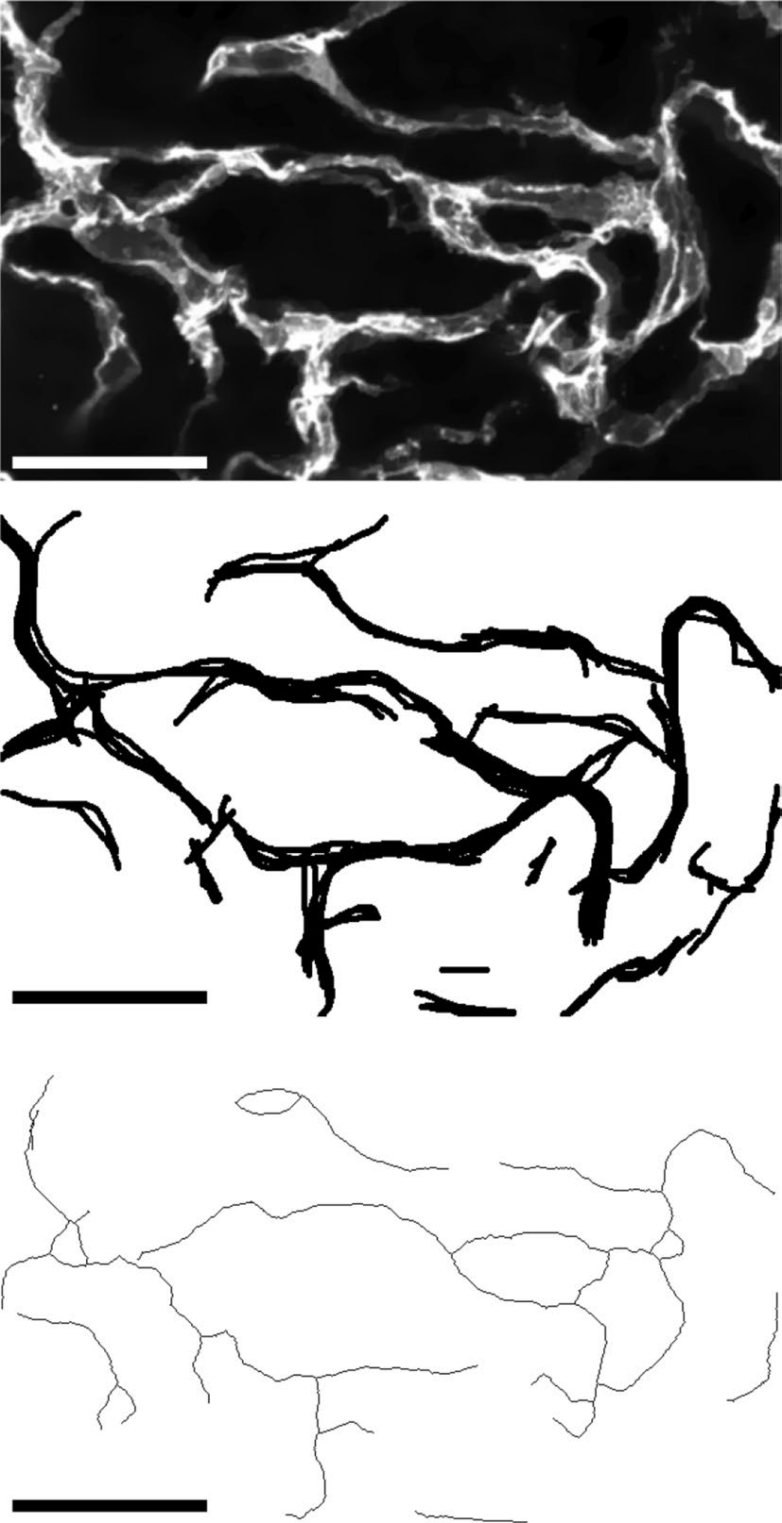
Exemplary cropped Z maximum projections of (from top to bottom) the fluorescence microscopy image of a blood vessel organoid, the ground truth, and the skeletonized image generated by VESNA based on Dataset A. Scale 50 $\upmu$m

We evaluated the segmentation quality achieved by VESNA using one example image. Specifically, we quantified the overlap between the skeletonized image produced by VESNA with a ground truth. For this analysis, we selected a section of an image of a blood vessel organoid (section “Generation of sample data”, Dataset A) and processed it with VESNA using parameter settings listed in Table S1. The raw image section, ground truth, binary and skeletonized image, and all measurement data returned by VESNA are available on the GitHub repository linked in section “Availability and requirements”. The ground truth was created by one expert who manually drew the ideal skeleton into each slice of the image stack (Fig. 5).

Comparison of the skeletonized image produced by VESNA and the ground truth yields an overlap of approximately 40% (Table 2). While this may initially seem to indicate low fidelity of the skeletonized image, this comparison is rather strict, as the skeletonized image is only one voxel wide, and the ground truth is rather narrowly defined. Because of this, even a slight deviation of the skeletonized image from the ground truth results in a lack of overlap.

**Fig. 6:**
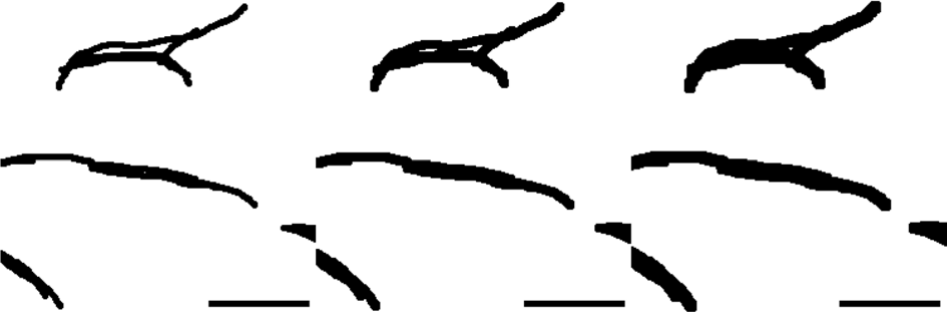
An exemplary cropped Z maximum projection of the ground truth of Dataset A: (from left to right) original, dilated once, and dilated twice. Scale 25 $\upmu$m

**Fig. 7:**
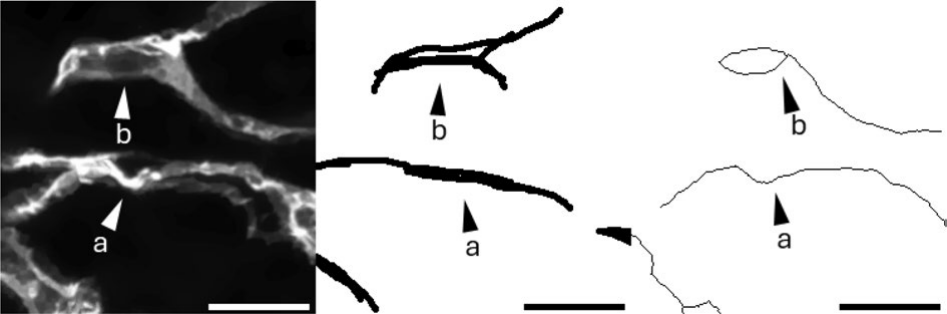
Exemplary cropped Z maximum projection of (from left to right) the raw image of a blood vessel organoid, the ground truth, and the skeletonized image of Dataset A. Marked is an example of a region where VESNA recognized a structure that is dislocated relative to the ground truth (**a**), and one where the segmented structures are more detailed than defined by the ground truth (**b**). Scale 25 ${\upmu }$m

To assess the level of divergence from the ground truth in a more detailed manner, the ground truth is dilated. Despite the apparent increase in area (Table 2), the dilation preserved most of the detail and definition of the original ground truth and still sets an appropriate level of stringency (Fig. 6). Comparison of VESNA’s returned skeletonized image with the dilated ground truth shows an increased overlap, reaching just over 60%. Further examination of the remaining discrepancies between the skeletonized image and the dilated ground truth reveals that VESNA often recognizes vascular segments in a way that matches the raw image, while the segments are displaced or simplified in the ground truth (Fig. 7a). Additionally, the nonhomogeneous fluorescence intensity within the vessels sometimes leads to an ambiguity in the network structure, where it is not objectively clear whether the ground truth or VESNA’s output depicts the structure more accurately (Fig. 7b).

**Table 2:** Percentages of skeleton voxels overlapping with the original and dilated ground truth, as well as area increase of the dilated ground truth relative to the original

Ground truth (area increase from original [%])	Overlap [%]
original	40.24
dilated once (49.31)	53.87
dilated twice (99.56)	61.85

We further recognized low-fluorescence structures in the original image. Many of these structures can be identified or completed by eye with relative ease, depending on the fluorescence intensity, but are sometimes not detected by VESNA. The adjustment of the brightness parameters as a way to recognize these structures leads to a loss of detail in finer and brighter fluorescence structures in this image. This implies a trade-off that is discussed further in section “Parameter Optimization”.

We conclude that even though the degree of overlap of VESNA’s segmentation with the ground truth appears moderate at first glance, a closer comparison of the skeletonized image, the ground truth and the raw image indicates a satisfactory result.

Next, we investigated the sensitivity of the overlap between the skeletonized image and the ground truth to the parameter settings of VESNA (Table S3). As a basis, we used parameter values that were optimized manually as detailed in section “Parameter Optimization”.

**Fig. 8:**
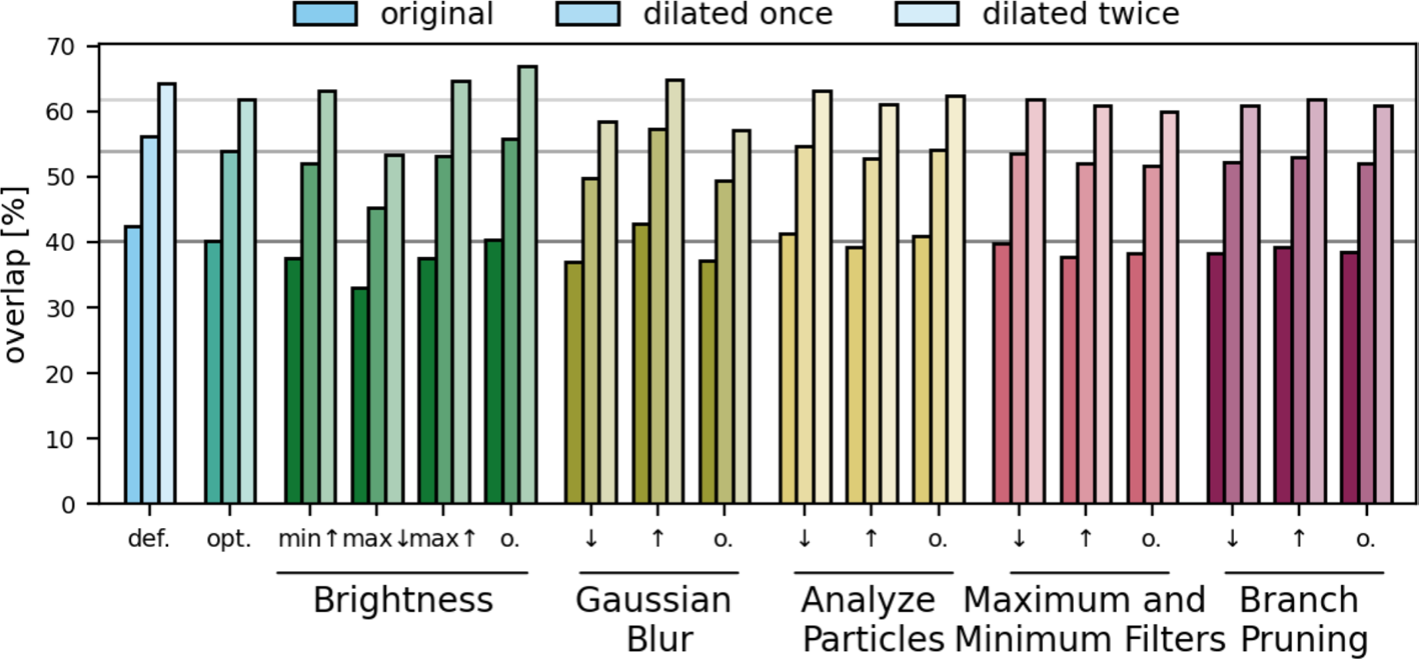
Percentages of skeleton voxels of Dataset A overlapping with the original and dilated ground truth at default (**def.**) and optimized (**opt.**) parameter settings, as well as varying values of each parameter (sample size $n=1$). Horizontal lines mark the overlap values with optimized parameter settings for easier comparison

The resulting percentages of overlapping skeleton voxels relative to the total skeleton voxels show that the deviations of the overlaps are small (Fig. 8). The most prominent decrease in overlap is caused by lowering the brightness maximum parameter. This setting leads to increased false positive segmentation, causing a higher number of skeleton voxels (7108 voxels compared to 4674 voxels resulting from optimized settings) and leading to the decreased percental overlap (32.96% with the original ground truth compared to 40.24%). The largest increase of the overlap is produced by a higher $\sigma$ value for the Gaussian blur. However, the effect is minimal (42.72% overlap with the original ground truth compared to 40.24% resulting from optimized settings).

The results demonstrate the robustness of the overlap between the resulting skeletonized image and the ground truth across varying parameter settings for this dataset.

Our results highlight the challenges of quantitatively evaluating segmented images generated by VESNA. Therefore, this process is not repeated for other raw images. Instead, we emphasize the significance of visually assessing the processed image data. In addition, we investigate the effect of the parameter settings on the network measurements.

### Sensitivity of network measurements

To determine the impact of parameter adjustments on the network measurement data, we performed a sensitivity analysis. A subset of the sample data that was found to be computationally viable for the high number of processing runs necessary for this analysis was selected (section “Generation of sample data”, Datasets C and D). The individual functions of the script are tested with parameter values chosen to be just outside the range that is typically found to be useful for most image data, as well as with the function omitted (Table S3). Notably, the actually useful range may vary.

The deviations between the network measurements generated from the tested parameter settings and the corresponding optimized settings are mostly not statistically significant (Figure S1). A notable exception is the segment length, which shows a significant deviation in 22 out of 34 parameter setting groups. This is easily explained by the large number of data points generated in this measurement. Given that each image contains hundreds of segments, which are measured and plotted individually, statistical significance is much more attainable.

**Fig. 9:**
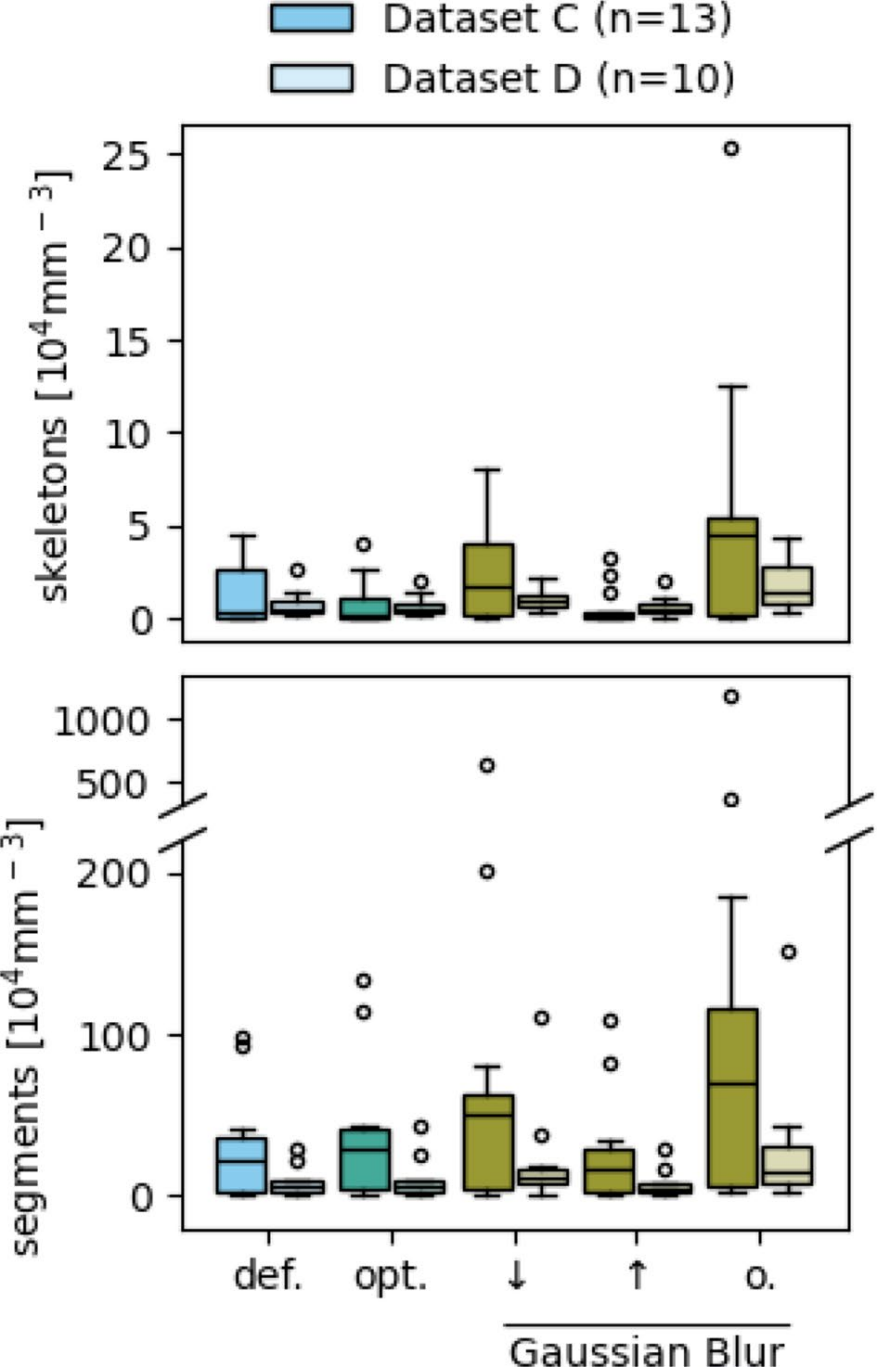
Comparison between the measurement data resulting from default (**def.**) and optimized (**opt.**) parameter settings, as well as from varying $\sigma$ values ($\pmb {\downarrow }$, $\pmb {\uparrow }$) and the omission (**o.**) of the Gaussian blur of Datasets C and D. None of the depicted data show a statistically significant deviation below a Bonferroni-corrected significance level of $\alpha = 0.0036$ from the optimized data. Complete data are shown in Figure S1

Although no significant deviations were observed in the remaining measurements, the effect sizes (Table S4) indicate several notable differences. These are generally expected and can be explained by considering each function’s purpose. For instance, a low $\sigma$ value or the omission of the Gaussian blur frequently results in failed or incomplete recombination of fragmented vessel structures. This, in turn, leads to an increased number of segments and skeletons, as seen in Fig. 9 and Table 3. Conversely, a high $\sigma$ value reconnects many of the fragmented vessel structures but leads to a loss of detail indicated by a decrease in segment and skeleton numbers.

**Table 3:** Effect sizes of the deviation of the numbers of skeletons and segments resulting from varying σ values (↓, ↑) and the omission (o.) of the Gaussian blur

Measurements		Glass’s $\Delta$		
		$\downarrow$	$\uparrow$	o.
Skeletons	C	**1.30**	*0.22*	**3.75**
	D	**0.53**	***0.13***	**1.99**
Segments	C	**1.46**	*0.25*	**3.35**
	D	**0.90**	*0.22*	**1.58**

Bold text indicates a high effect, italic a medium and bolditalic a low effect size. Complete set of effect sizes is shown in Table S4

Our results show that certain parameters strongly affect the network measurements (Table S4) and, therefore, require special attention during parameter adjustment. Parameters with a strong effect are, for example, the $\sigma$ value for the Gaussian blur, the brightness settings, and the Branch Pruning threshold. In contrast, variations in other parameters, such as the Analyze Particles threshold and the maximum and minimum filters, result in less deviation from the optimized data.

**Fig. 10:**
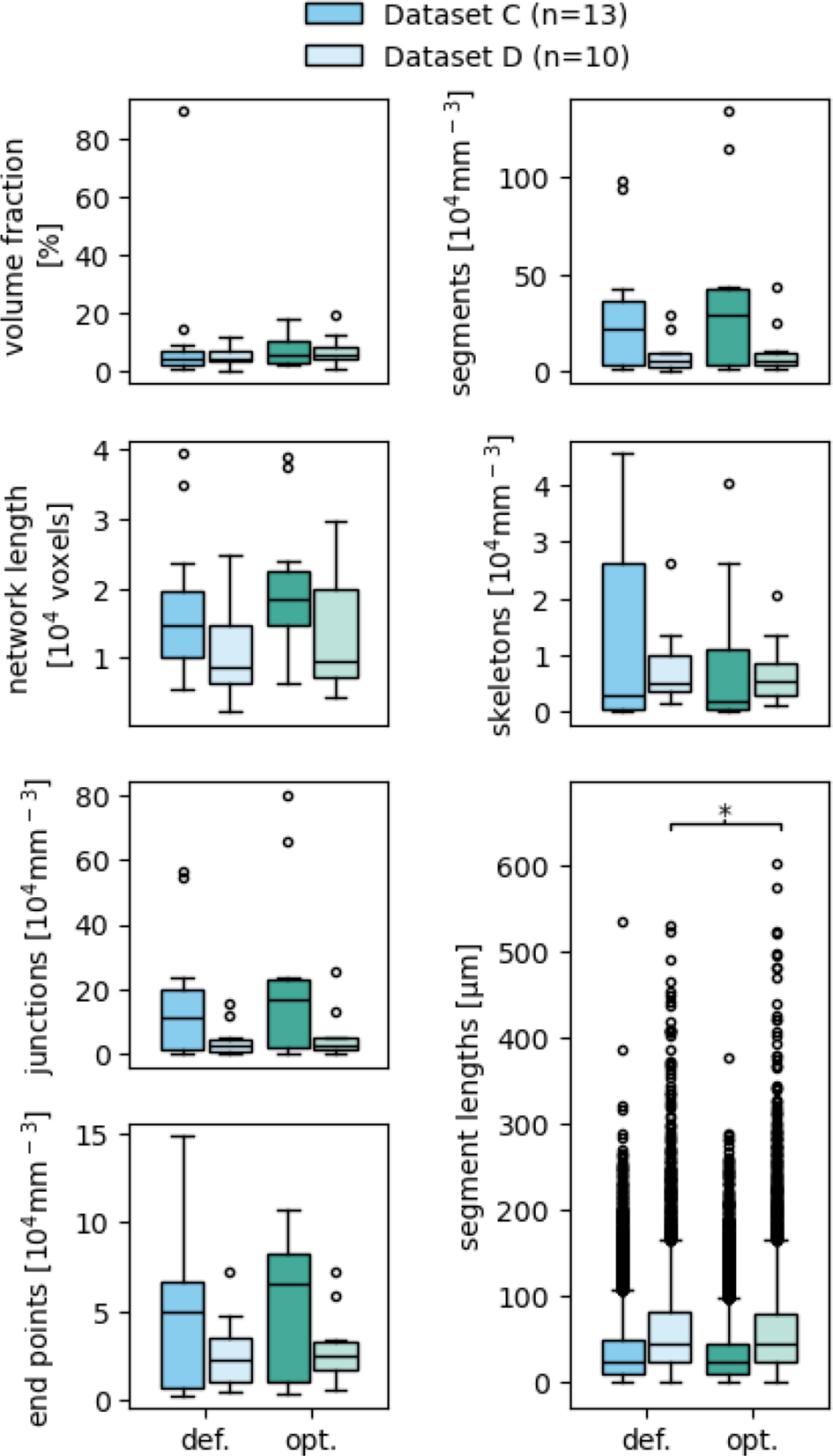
Comparison between the measurement data resulting from default (**def.**) and optimized (**opt.**) parameter settings. Boxes that are marked with an asterisk (*) show a statistically significant deviation for a significance level of $\alpha = 0.05$ between the default and optimized parameter settings, based on a two-sided Mann–Whitney U test. Complete data are shown in Figure S1

The network measurement data generated with default settings are mainly consistent with those produced with the optimized settings, except for the volume fraction measurement (Fig. 10, Table 4). This suggests that the default settings can reasonably be used for preliminary screening. However, the process should always be controlled by visual comparison of the returned binary and skeletonized images to the raw image.

**Table 4:** Effect sizes of the deviation of the measurement data resulting from the default parameter settings from those resulting from optimized parameter settings

Measurements	Dataset	Glass's ∆
Volume fraction	C	0.94
	D	*0.39*
Network length	C	*0.28*
	D	*0.29*
Skeletons	C	*0.31*
	D	***0.13***
Segments	C	*0.22*
	D	***0.17***
Junctions	C	*0.22*
	D	***0.18***
End points	C	***0.19***
	D	***0.19***
Segment lengths	C	***0.07***
	D	***0.03***

Bold text indicates a high effect, italic a medium, and bolditalic a low effect size. Complete set of effect sizes are shown in Table S4

### Application to sample data

#### Effect of anti-angiogenic drug on vasculature

To demonstrate a possible application of VESNA, we used it to assess the effect of sorafenib on the vascular networks generated in blood vessel organoids.

Sorafenib is a tyrosine kinase inhibitor. One of its many functions is suppressing the activation of the vascular endothelial growth factor receptors VEGFR-1 and -2 that are necessary for vascular development, vessel integrity, and cell contacts [32]. The deregulation of the VEGF/VEGFR system induces neoangiogenesis, one of the hallmarks of cancer. This allows for increased nutrient supply to the tumor cells and is associated with increased growth and dissemination of the tumor [32, 33]. Sorafenib has been approved for the treatment of some carcinomas by the FDA in 2005 [34] and by the EMA in 2006 [35] for its angiogenic effect. We used VESNA to investigate the change in network structure induced by sorafenib.

The acquired images of the drug-treated and control groups (section “Generation of sample data”, Dataset B) are processed using our macro. Due to the large dimensions, the raw images are scaled by a factor of 0.5 in the x and y dimensions prior to processing. This greatly reduces the necessary processing power and time. The scaling is not expected to lead to a relevant loss of information in the case of this dataset, as the generated vascular networks show sufficiently low detail. In one image, part of a second organoid was included in the imaged volume and was manually removed from the image stack. The parameters were iteratively adjusted by visual evaluation of the resulting binary and skeletonized images. The final parameter settings can be found in Table S1. Two of the available images of drug-treated organoids were not able to be properly processed, even after parameter adjustment, due to high background fluorescence and low fluorescence intensity of the vascular tissue. These images were omitted from further data analysis.

**Fig. 11:**
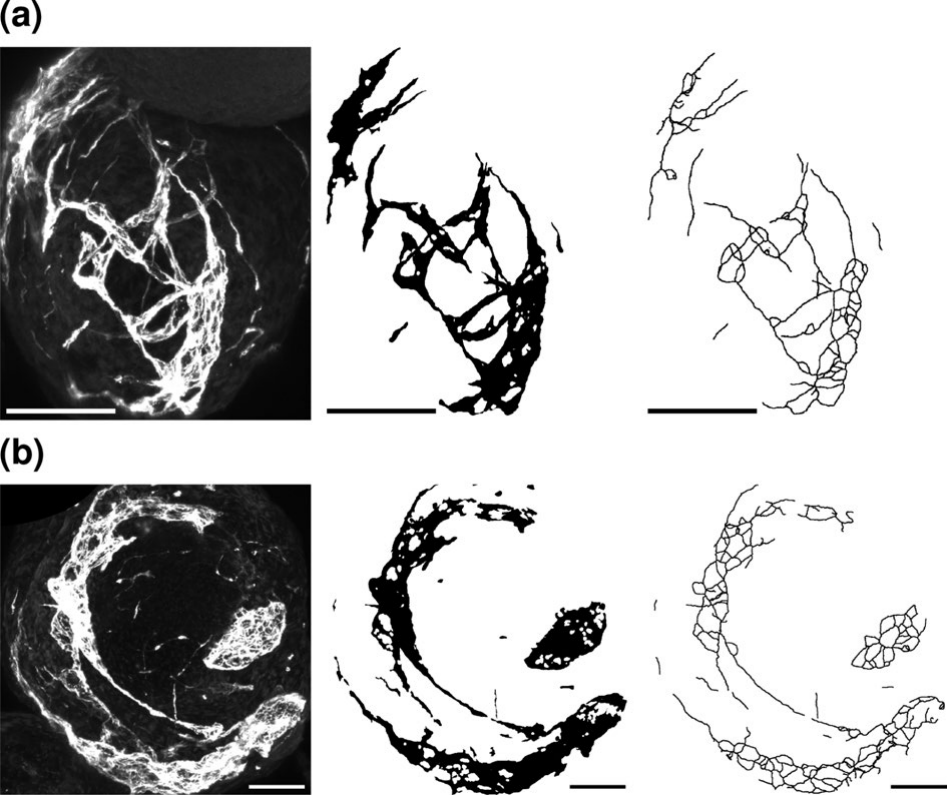
Exemplary cropped Z maximum projections of blood vessel organoids of the control group (**a**) and the group treated with 15 nM sorafenib (**b**) of Dataset B. Depicted are (from left to right) raw images, binary images, and skeletonized images. Skeletonized images are dilated for better visibility. Scale 100 $\upmu$m

Organoids of the control group and the drug-treated group are depicted in Fig. 11a and 11b, respectively. The control group organoid shows a vascular network that is distributed throughout most of the organoid. In contrast, the drug-treated organoid lacks vasculature in a large central area. This observation already aligns with the antiangiogenic effect of sorafenib treatment. The size difference between the two organoids can likely be attributed to random variation, given the considerable heterogeneity of organoid sizes throughout the experiment.

**Fig. 12:**
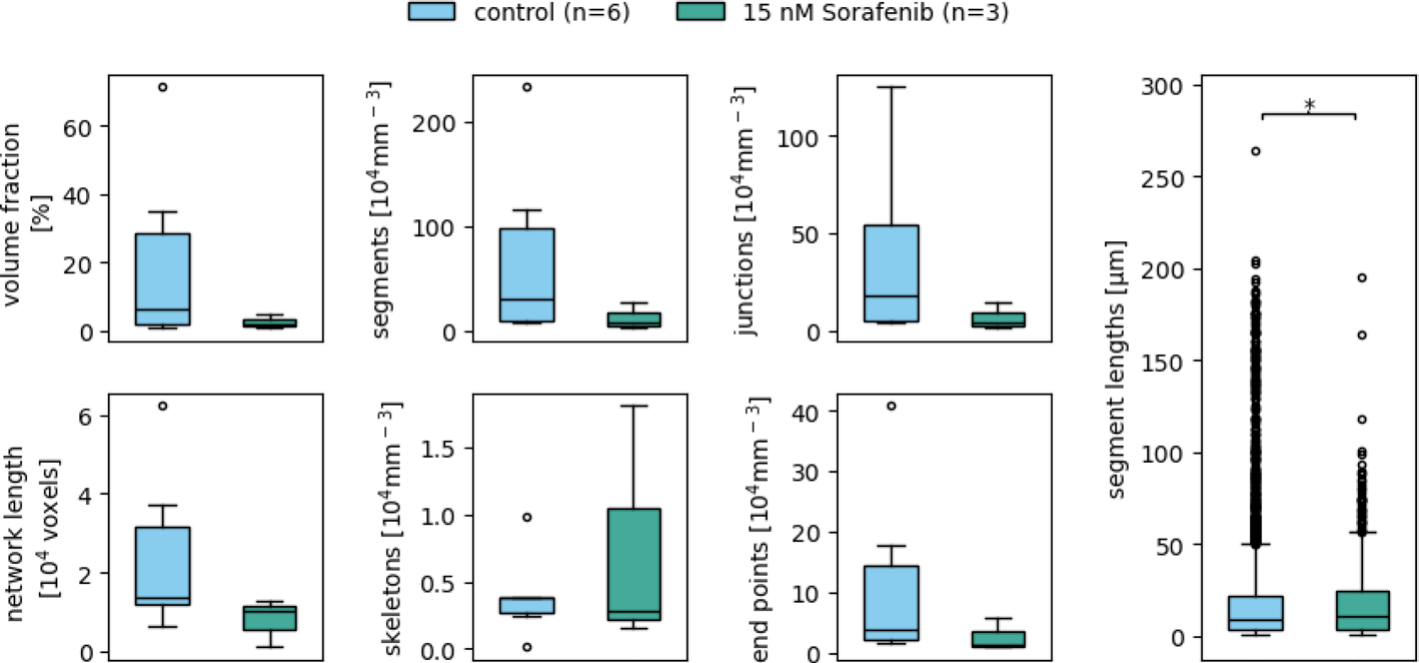
Network measurement data resulting from the drug-treated and control groups of Dataset B. The utilized parameter values are listed in Table S1. Boxes that are marked with an asterisk (*) show a statistically significant deviation for a significance level of $\alpha = 0.05$

The resulting network measurement data (Fig. 12) show a decrease in all measurements, except for the number of skeletons. While the measurements do not show a statistically significant deviation, in part due to the low numbers of images per group ($n = 6$ for the control group, $n = 3$ for the drug-treated group), the associated Glass’s $\Delta$s (Table 5) suggest that sorafenib has a considerable effect on the structure of the vascular networks. The networks seem to be looser and less complex under drug treatment conditions, as indicated by decreased numbers of junctions and end points and decreased network density. Notably, while the segment length diverges in a statistically significant way, the effect size is small in comparison to the other measurements. This can be explained by the high number of data points generated for this measurement, which favors a statistical significance.

**Table 5:** Effect sizes of the deviation of the different network measurement data resulting from the drug-treated blood vessel organoids compared to the control group of Dataset B

Measurements	Glass’s $\Delta$
Volume fraction	**0**.**69**
Network length	**0**.**82**
Skeletons	**1**.**20**
Segments	**0**.**72**
Junctions	**0**.**73**
End points	**0**.**63**
Segment lengths	***0.04***

Bold text indicates a high effect, italic a medium, and bolditalic text a low effect

These observations coincide with the exemplary images (Fig. 11). Considering that the measurements are normalized to the imaged volume, the images of the drug-treated organoids show fewer skeletons, junctions, and end points than the control group organoid.

The presented results are consistent with the above-mentioned biochemical functions of sorafenib as an antiangiogenic agent and demonstrate the possible application of our macro VESNA to biomedically relevant experimental setups.

#### Effect of growth conditions on vasculature

Another possible application of VESNA is analyzing the effect of different growth conditions of organoids on the vascular network. For this, organoids were generated using smaller and larger molds. The exact growth conditions are detailed in section “Blood vessel organoid generation”, Dataset C. For the processing of the images, only the brightness parameters are adjusted to accommodate for the low fluorescence intensity of the original images. The parameter settings can be found in Table S1.

**Fig. 13:**
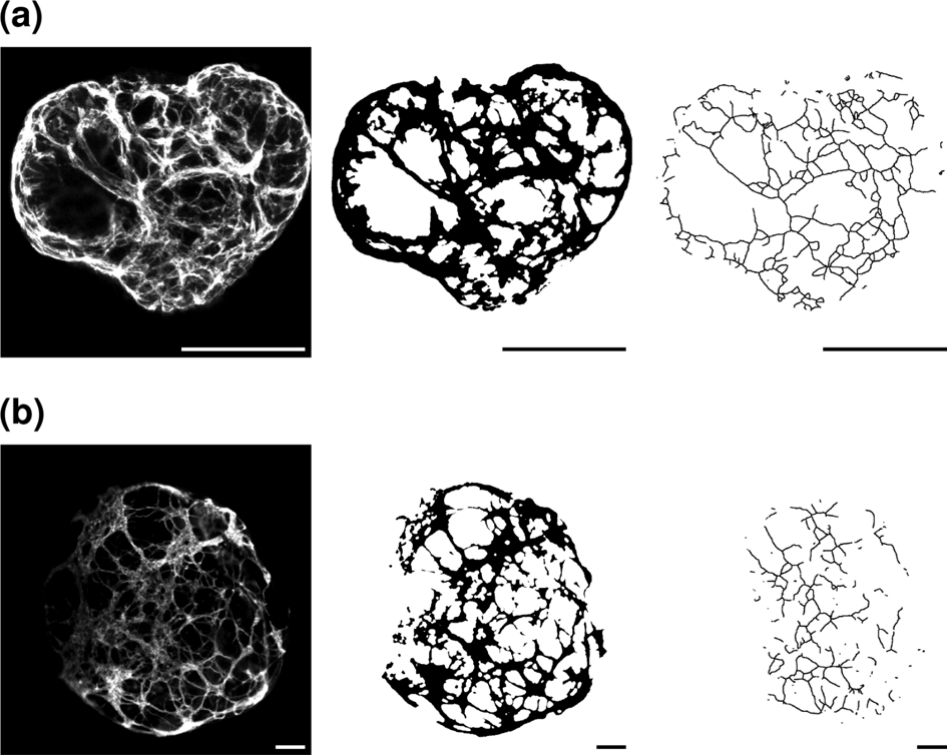
Exemplary cropped Z maximum projections of blood vessel organoids of Dataset C seeded in small molds (**a**) and large molds (**b**). Depicted are (from left to right) raw images, binary images, and skeletonized images. Skeletonized images are dilated for better visibility. Scale 100 $\upmu$m

Exemplary sections of the images resulting from both groups are depicted in Fig. 13. Taking the scales of the images into consideration, the vascular structure of the organoid generated from a small mold (Fig. 13a) is smaller and more detailed. On the other hand, the structure generated from seeding in the larger mold (Fig. 13b) is looser and less complex.

**Fig. 14:**
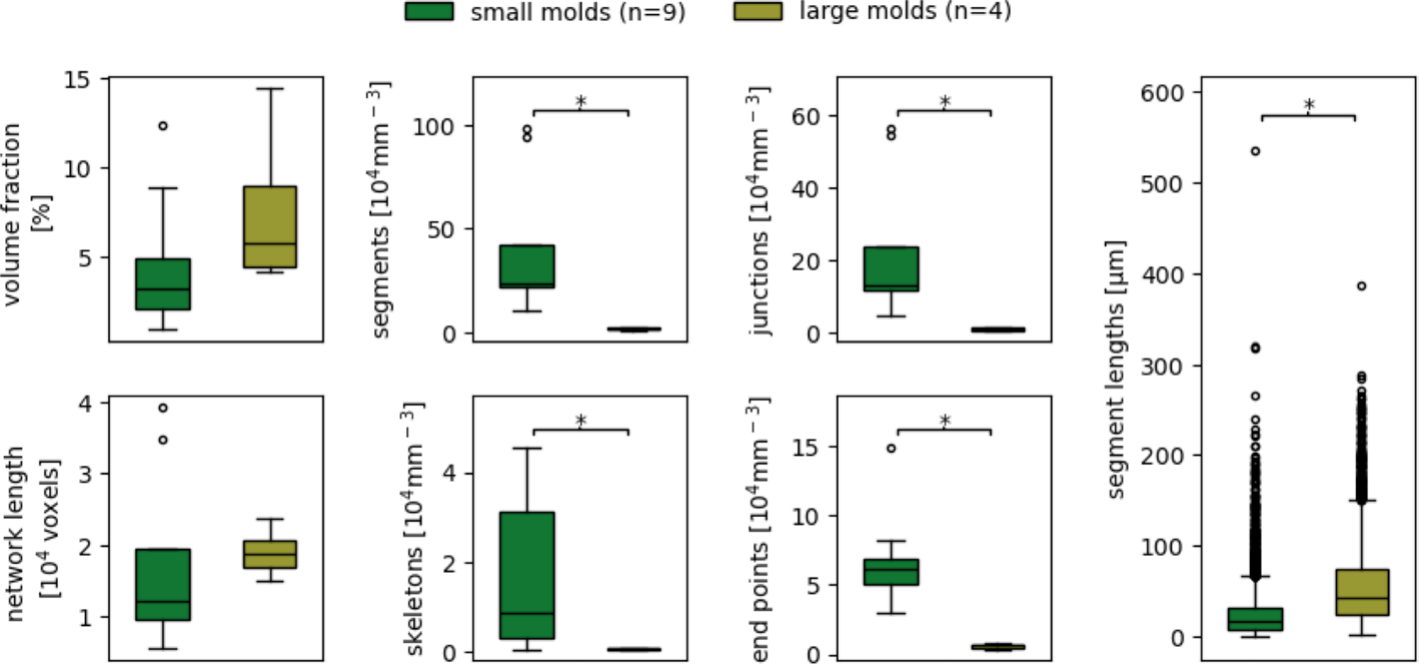
Complete measurement data resulting from the two different mold size groups of Dataset C. The utilized parameter values are documented in Table S1. Boxes that are marked with an asterisk (*) show a statistically significant deviation for a significance level of $\alpha = 0.05$

Plotted network measurement data allow a closer look at the difference in structure between both groups of vascular networks. Even at the low number of data used ($n = 9$ for small molds, $n = 4$ for big molds), five out of the seven measurements show statistically significant deviations (Fig. 14) and high effect sizes (Table 6). While seeding of the organoids in small molds creates more complex vascular networks with high numbers of segments, junctions, and end points, they are also more fragmented, as indicated by the increased number of skeletons. The usage of larger molds leads to simpler networks with fewer but, on average, longer segments. The network length and volume do not differ much. These observations are also reflected in the exemplary image sections (Fig. 13).

**Table 6:** Effect sizes of the deviation of the different network measurement data resulting from the different mold sizes of Dataset C

Measurements	Glass’s $\Delta$
Volume fraction	**0**.**87**
Network length	***0.18***
Skeletons	**1**.**24**
Segments	**1**.**10**
Junctions	**1**.**19**
End points	**1**.**84**
Segment lengths	**1**.**19**

Bold text indicates a high effect, italic a medium, and bolditalic text a low effect

#### Application to hydrogel-based cultures

Because of the simplicity of the experimental setup required, the usage of our macro VESNA is not limited to microscopy images of organoids, but can easily extend to other three-dimensional culturing methods. To demonstrate this, we processed and analyzed images of three-dimensional hydrogel-based blood vessel cultures (section “Generation of bioprinted hydrogel-based vascular cultures”, Dataset D).

The default parameters were acceptable for the processing of the hydrogel culture images for the most part, only requiring optimization in the brightness parameters (Table S1).

**Fig. 15:**
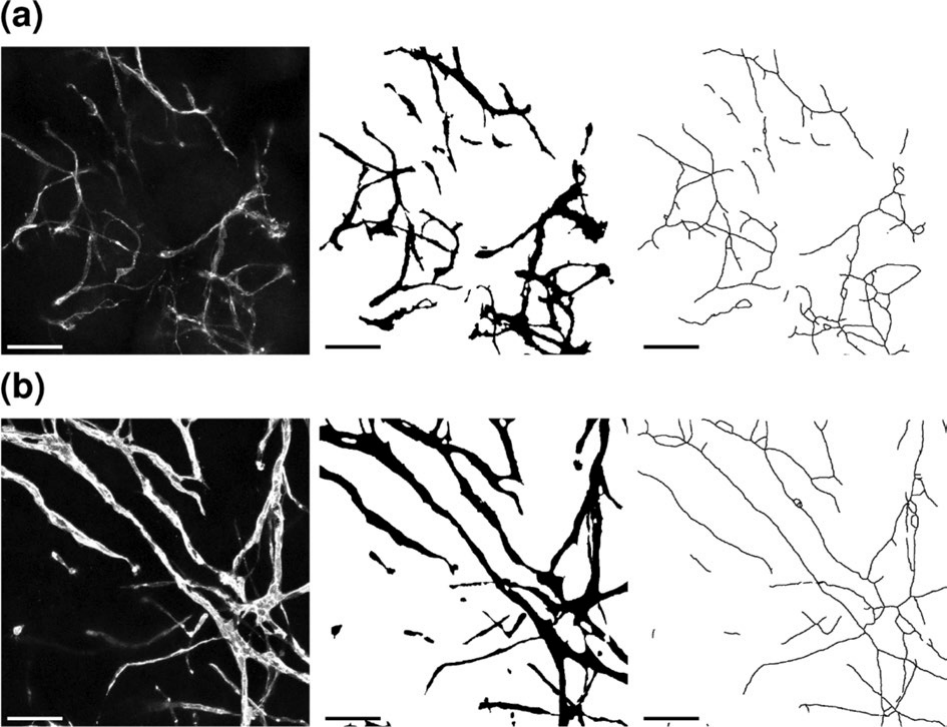
Exemplary cropped Z maximum projections of hydrogel-based blood vessel cultures of Dataset D at days 7 (**a**) and 17 (**b**). Depicted are (from left to right) raw images, binary images, and skeletonized images. Skeletonized images are dilated for better visibility. Scale 100 $\upmu$m

The exemplary image sections show a vascular network at day 7 (Fig. 15a) that consists of a majority of shorter segments. The older network at day 17 (Fig. 15b) is mainly made up of longer segments, but there are still a number of small segments to be found. Notably, the segments in the structure at day 7 seem to be randomly oriented in all directions, while the vessels at day 17 show a more parallel arrangement (Fig. 15).

**Fig. 16:**
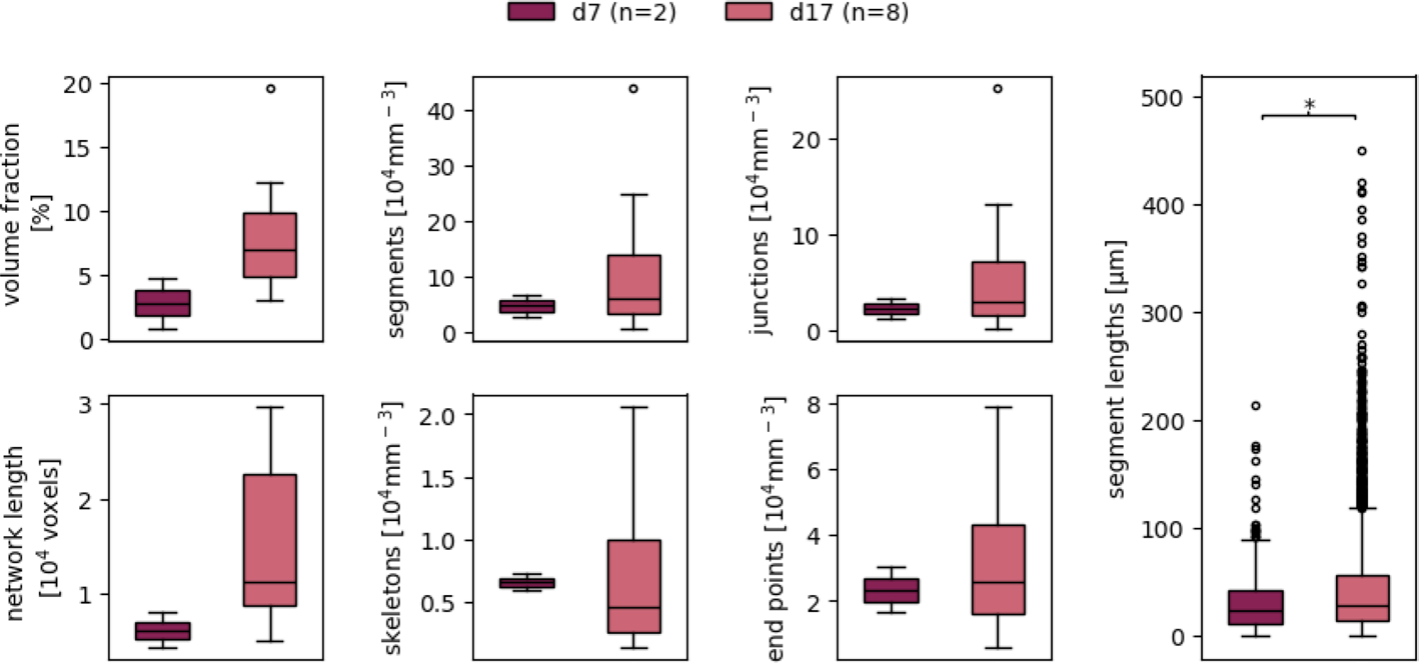
Complete network measurement data resulting from hydrogel cultures of Dataset D at days 7 and 17. The utilized parameter values are documented in Table S1. Boxes that are marked with an asterisk (*) show a statistically significant deviation for a significance level of $\alpha = 0.05$

The limited sample size at day 7 ($n = 2$) likely accounts for the absence of statistical significance in most measurements and may also contribute to the relatively high effect sizes observed (Fig. 16, Table 7). Despite this, the data suggest growth in vascular networks between days 7 and 17, as evidenced by increased segment lengths. While the median segment length remains similar, vascular structures at day 17 exhibit an increased upper limit of segment lengths. This growth is further supported by elevated counts of skeletons, segments, junctions, and end points, along with increases in vascular length and volume fraction, all of which are also observed in the exemplary images (Fig. 15).

**Table 7:** Effect sizes of the deviation of the different network measurement data resulting from hydrogel-based cultures of Dataset D at days 7 and 17

Measurements	Glass’s $\Delta$
Volume fraction	**2**.**85**
Network length	**4**.**85**
Skeletons	**3**.**62**
Segments	**1**.**11**
Junctions	**3**.**40**
End points	**1**.**36**
Segment lengths	*0.36*

Bold text indicates a high effect, italic a medium, and bolditalic text a low effect

Our results highlight the versatility of VESNA in facilitating quantitative analyses of vascular networks in organoids and hydrogel-based cultures across diverse research applications, including studies on the effect of drug mechanisms and growth conditions.

## Discussion

### VESNA’s performance

In-depth comparison of a generated skeletonized image to a manually labeled ground truth demonstrates high quality and fidelity of VESNA’s segmentation and skeletonization, with some weaknesses observed in regions exhibiting low signal intensity. Quantitative analysis of the overlap of the skeletonized image with the ground truth is particularly sensitive in this context, given the thinness of the skeletonized structures. Despite this sensitivity, the overlap between the skeletonized image and the ground truth proves robust across a range of parameter settings. The adjustment of the $\sigma$ value for the Gaussian blur and the brightness settings yields the largest effects, even though they are still small.

Most network measurements remain consistent and reliable under varying parameter values. High effects of parameter adjustment are observed for the $\sigma$ value of the Gaussian blur, the brightness settings, and the Branch Pruning threshold, indicating that these parameters need to be selected carefully. Smaller effects are observed for parameters such as the Analyze Particles threshold and the maximum and minimum filters. Through this analysis, we underlined the robustness of our macro and identified the most critical parameters for optimizing the segmentation and measurement process, providing guidance for applications of VESNA.

The optimized parameter settings yield network measurement data comparable to that obtained with default settings. This suggests that fully automated processing using the default parameters is feasible for data sets with fluorescence intensity, background fluorescence, and level of detail comparable to our tested data. The possibility to bypass the parameter optimization process, the most time-intensive part of the application for the user, further enhances the macro’s applicability and usability across diverse research scenarios.

### Applications

By demonstrating VESNA’s performance across three different applications, we provide evidence for its robustness across different image data and experimental objectives. First, we tested the effect of sorafenib on the vascular structure generated in blood vessel organoids. Among other mechanisms of action, sorafenib inhibits the activation of VEGFRs, which are responsible for proliferation, angiogenesis, and vascular sprouting, explaining the drug’s anti-angiogenic effect [32, 33]. This is consistent with our observed results, showing reduced network size and complexity, as indicated by a decrease in junction and end points, as well as the number of skeletons compared to the control group. Kumar et al. [36] were able to show similar results in a 2D chick chorioallantoic membrane-based experiment. They observed a decrease in network length, branch length, and junction point counts in sorafenib-treated chicken embryos compared to the control group. In opposition to our results, they have also observed a decrease in branch numbers, whereas we observed an increase.

We have also tested the effect of different growth conditions on the vascular networks and found that organoids generated from smaller molds develop more complex but also more fragmented vascular structures as evidenced by increased numbers of segments, junction and end points, and skeletons. An alternative explanation for the difference in measurements might be the different staining behavior of larger organoids, which is known to be a potential cause of decreased signal intensity towards the center of the organoid [37]. This low fluorescence intensity is also present in our corresponding sample image (Fig. 13b), supporting the existence of this effect in our case. But while this effect might seem like a reasonable alternative explanation, the sample image also shows that our macro is able to successfully recognize these low-intensity structures. Instead, a likely explanation for the different vascular structures resulting from different growth conditions might be very similar: Analogous to how fluorophore-coupled antibodies are limited in their diffusion to the center of a large organoid, leading to lower signal intensity, VEGF is likely also diffusion-limited to some degree. This would result in an effectively lower concentration of VEGF in the center of larger organoids compared to smaller organoids, restricting the level of activation of VEGFRs and the resulting angiogenesis.

Finally, we demonstrate the ability of our macro to process images generated from other 3D tissue culturing methods, with an example of hydrogel-based cultures. In this experiment, we observe an increase in all measurements as the vascular network grows from day 7 to 17, as expected. The current version of our macro does not include a representation of vessel direction, which limits its ability to distinguish between random and statistically significant observations in this case. This highlights a possible area of further improvement for our macro. The application to other tissue cultures is enabled by the simplicity of our macro’s requirements towards the input images. Specifically, it only requires a vasculature-specific stain, such as CD31, and in the case of larger objects, tissue clearing is also necessary. This provides flexibility that not only makes the processing of a wide range of blood vessel cultures possible, but, given an appropriate antibody target, makes the processing of lymphatic networks and other vascular-like networks plausible.

Collectively, these three example applications demonstrate the robust usability of VESNA across different experimental goals, as well as across a diverse range of image data generated from different tissue culturing methods and varying in features such as age, network size and complexity, fluorescence intensity, background noise, as well as image size. All three datasets are successfully processed after minor parameter adjustment, indicating an even wider range of possible image data than shown here.

Additionally, VESNA can be readily adapted to diverse image data and additional research applications by modifying the pre- and post-processing steps to the specific image data at hand or by leveraging Fiji’s extensive library of macros and plugins to incorporate additional measurements.

## Conclusion

VESNA addresses a central gap in vascular image analysis by providing a fully automated pipeline for quantification of three-dimensional *in vitro* vascular networks from fluorescence microscopy data. VESNA is designed for Fiji, a well-established open-source software, making it simple to use for researchers with limited image analysis experience, while still easily modifiable for diverse research questions. With these features, VESNA represents a valuable tool for research in vascular development, disease mechanisms, drug discovery, and related applications.

## Availability and requirements

Project name: VESNA (Vessel Segmentation and Network Analysis)

Project home page: https://github.com/scfischer/schuettler-et-al-2025

Operating systems: Windows, MacOS, Linux

Programming languages: ImageJ macro, BeanShell

Other requirements: Fiji (ImageJ), Bio-Formats and IJPB-plugins Update Sites

License: MIT License

Any restrictions to use by non-academics: None

## Supplementary Information

Below is the link to the electronic supplementary material.

Supplementary Material.

## Data Availability

The macro together with instructions and a sample image are available on https://github.com/scfischer/schuettler-et-al-2025/ (archived for publication: 10.5281/zenodo.17019176).

## Abbreviations

FCS: Fetal calf serum
hESC: Human embryonic stem cell
hiPSC: Human induced pluripotent stem cell
MIM: Mesodermal induction medium
MPC: Mesodermal progenitor cell
NGS: Normal goat serum
OMM: Organoid maintenance medium
P/S: Penicillin/Streptomycin
PFA: Paraformaldehyde
TZ: Thiazovivin
VEGF: Vascular endothelial growth factor
VEGFR: Vascular endothelial growth factor receptor
VGM: Vascular growth medium
