# Supplementary material for: VESNA: an open-source tool for automated 3D vessel segmentation and network analysis
Source: BMC Bioinformatics. 2025 Oct 21;26:254. doi: 10.1186/s12859-025-06270-6 (PMC12539100; doi:10.1186/s12859-025-06270-6)
Supplement: Supplementary file 1 — Supplementary Material. [file 12859_2025_6270_MOESM1_ESM.pdf]

Appendix

Table S1. Default parameter values preset in the macro and chosen values for processing of each set of sample data. Dashes (**-**) indicate no deviation from default values.

parameters	default	Dataset A	Dataset B	Dataset C	Dataset D
brightness min	0	-	-	1	1
brightness max	120	110	255	50	50
Gaussian blur σ [voxels]	1.8	-	2.0	-	-
size threshold [pixels]	55	-	-	-	-
maximum filter [pixels]	3	-	-	-	-
minimum filter [pixels]	4	-	-	-	-
pruning length [voxels]	20	-	-	-	-

Table S2. Dimensions of all processed images by dataset, as well as observed processing times for some exemplary images at optimized parameter settings. All dimensions are rounded to five significant places. All images of Dataset B were rescaled prior to processing, as described in Section 3.3. Listed are dimensions and voxel sizes of the rescaled images.

dataset	image dimensions [px]			voxel size [µm]			image dimensions [µm]			processing time
	X	Y	Z	X	Y	Z	X	Y	Z	
Dataset A	646	398	170	0.31074	0.31074	0.95000	200.74	123.67	161.50	30 sec
Dataset B	761	963	210	0.38221	0.38221	0.95000	290.87	368.07	199.50	
	846	990	237	0.62148	0.62148	0.95000	525.77	615.27	225.15	
	944	879	156	0.32624	0.32624	0.95000	307.97	286.76	148.20	
	978	702	100	0.27969	0.27969	0.95000	273.82	196.35	95.00	
	929	941	179	0.29028	0.29028	0.95000	269.67	273.15	170.05	
	1024	1024	281	0.54998	0.54998	0.95000	563.18	563.18	266.95	
	1024	1024	220	0.62148	0.62148	0.95000	636.40	636.40	209.00	3 min
	201	222	177	1.6934	1.6934	0.95000	340.38	375.94	168.15	<10 sec
	767	894	163	0.47369	0.47369	0.95000	363.32	423.48	154.85	
Dataset C	1024	1024	54	1.1713	1.1713	5.0000	1199.39	1199.39	270.00	
	1024	1024	57	1.4326	1.4326	5.0000	1467.03	1467.03	285.00	
	1024	1024	72	2.1654	2.1654	5.0000	2217.41	2217.41	360.00	
	1024	1024	80	1.4514	1.4514	4.0000	1486.21	1486.21	320.00	
	1024	1024	28	0.62148	0.62148	5.0000	636.40	636.40	140.00	
	1024	1024	43	0.33057	0.33057	2.0000	338.51	338.51	215.00	
	1024	1024	112	0.62148	0.62148	0.95000	636.40	636.40	106.40	2 min
	1024	1024	35	0.62148	0.62148	0.95000	636.40	636.40	33.24	
	1024	1024	48	0.62148	0.62148	0.95000	636.40	636.40	45.60	
	1024	1024	71	0.62148	0.62148	0.95000	636.40	636.40	67.45	
	1024	1024	17	0.28033	0.28033	4.0000	287.05	287.05	68.00	
	1024	1024	56	0.46379	0.46379	3.0000	474.92	474.92	168.00	
	1024	1024	40	0.31074	0.31074	2.0000	318.20	318.20	80.00	
Dataset D	1024	1024	30	0.62148	0.62148	5.0000	636.40	636.40	150.00	
	1024	1024	15	0.62148	0.62148	5.0000	636.40	636.40	75.00	
	1024	1024	25	0.62148	0.62148	3.0000	636.40	636.40	75.00	
	1024	1024	24	0.62148	0.62148	1.0000	636.40	636.40	24.00	
	1024	1024	18	0.62148	0.62148	3.0000	636.40	636.40	54.00	
	1024	1024	11	0.62148	0.62148	2.5000	636.40	636.40	27.50	
	1024	1024	30	0.62148	0.62148	3.0000	636.40	636.40	90.00	
	1024	1024	26	0.62148	0.62148	4.0000	636.40	636.40	104.00	
	1024	1024	24	3.1074	3.1074	7.0000	3181.98	3181.98	168.00	
	1024	1024	33	3.1074	3.1074	7.5000	3181.98	3181.98	247.50	

Additional Data on Sensitivity of Parameters

Table S3. Parameter values used for the processing of the images of Datasets A, C and D in the parameter sensitivity test. The selected parameter values are chosen to lie just outside of the value range that was found to be useful for most datasets. Dashes (**-**) indicate no deviation from optimized values, while **o.** indicates the omission of the function. *For Dataset A, a brightness maximum of 110 is utilized.

			**Brightness**				**Gaussian blur**			**Analyze Particles**			**Max. and Min. filters**			**Branch Pruning**		
**parameters**	**def.**	**opt.**	**min ↑**	**max ↓**	**max ↑**	**o.**	**↓**	**↑**	**o.**	**↓**	**↑**	**o.**	**↓**	**↑**	**o.**	**↓**	**↑**	**o.**
brightness min	0	1	30	-	-	o.	-	-	-	-	-	-	-	-	-	-	-	-
brightness max	120	50*	-	30	200	o.	-	-	-	-	-	-	-	-	-	-	-	-
Gaussian blur σ [voxels]	1.8	1.8	-	-	-	-	0.5	3.0	o.	-	-	-	-	-	-	-	-	-
size threshold [pixels]	55	55	-	-	-	-	-	-	-	20	150	o.	-	-	-	-	-	-
maximum filter [pixels]	3	3	-	-	-	-	-	-	-	-	-	-	1	6	o.	-	-	-
minimum filter [pixels]	4	4	-	-	-	-	-	-	-	-	-	-	1	7	o.	-	-	-
pruning length [voxels]	20	20	-	-	-	-	-	-	-	-	-	-	-	-	-	10	100	o.

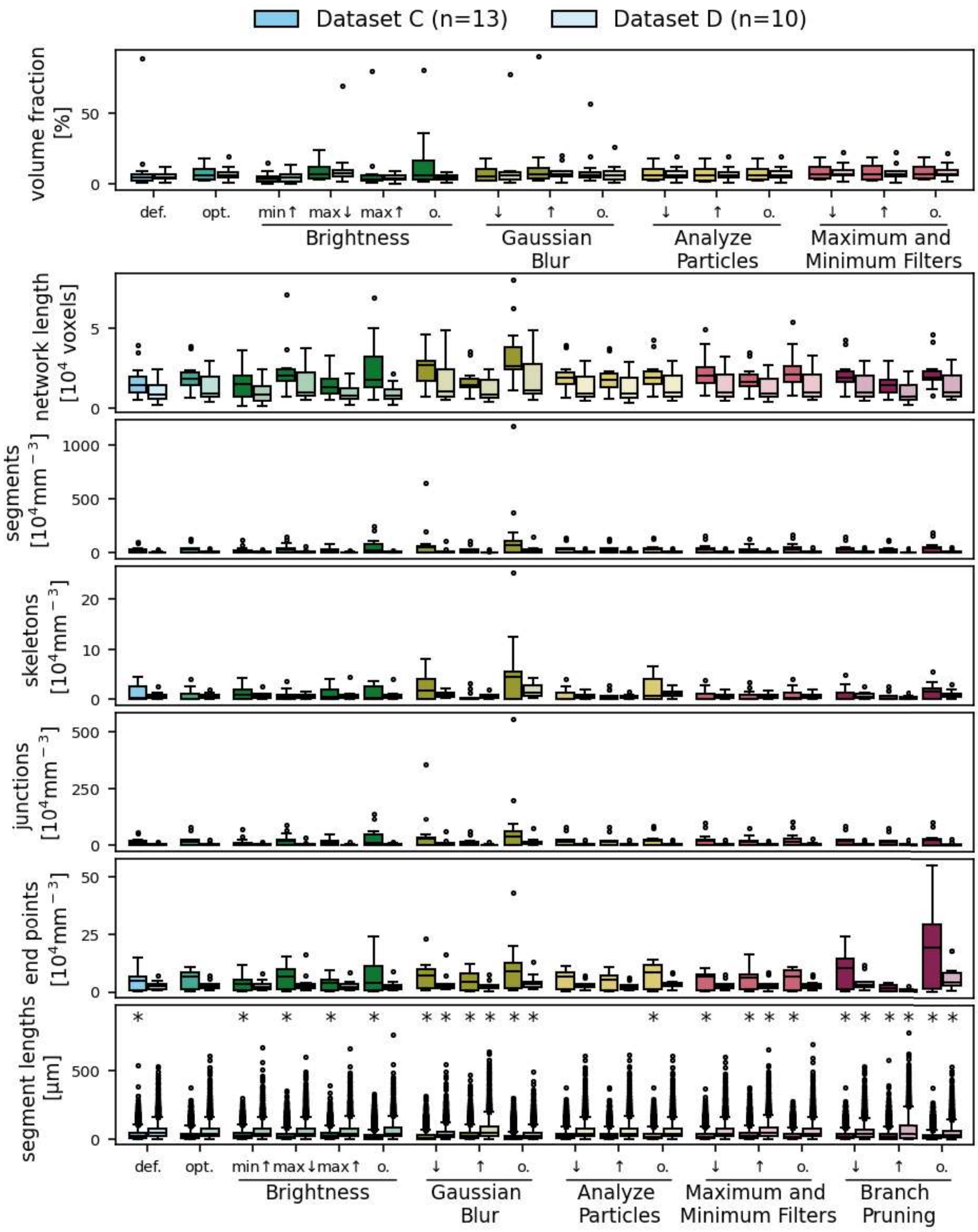

Figure S1. Complete measurement data resulting from the parameter sensitivity test performed on images of Datasets C and D. The utilized parameter values are documented in Table S3. Boxes that are marked with an asterisk (*) show a statistically significant deviation from the optimized measurement data, using a Bonferroni-corrected significance level $\alpha = 0.0036$ for the volume fraction measurement and $\alpha = 0.0029$ for the remaining measurements.

Table S4. Effect sizes (rounded to two decimal places) of the deviation of the resulting measurement data of tested parameter settings from the optimized settings. **Red** text color indicates a high effect, **green** a medium, and **blue** text color a low effect. **C**, **D**: Datasets C and D.

measurements		def.	Brightness min ↑	Brightness max ↓	Brightness max ↑	Brightness o.	Gaussian blur ↓	Gaussian blur ↑	Gaussian blur o.	Analyze Particles ↓	Analyze Particles ↑	Analyze Particles o.	Max. and Min. filters ↓	Max. and Min. filters ↑	Max. and Min. filters o.
volume fraction	C	0.94	0.58	0.46	0.62	1.93	0.06	1.45	0.83	0.01	0.03	0.02	0.24	0.27	0.22
	D	0.39	0.46	1.19	0.55	0.54	0.99	0.20	0.09	0.01	0.03	0.02	0.28	0.19	0.27
network length	C	0.28	0.54	0.43	0.53	0.58	0.58	0.30	1.47	0.05	0.10	0.12	0.38	0.20	0.45
	D	0.29	0.35	0.33	0.43	0.41	0.60	0.18	0.66	0.02	0.04	0.03	0.13	0.07	0.15
segments	C	0.22	0.30	0.19	0.32	0.60	1.46	0.25	3.35	0.04	0.05	0.11	0.18	0.12	0.24
	D	0.17	0.17	0.19	0.25	0.21	0.90	0.22	1.58	0.03	0.03	0.07	0.04	0.05	0.07
skeletons	C	0.31	0.29	0.05	0.12	0.27	1.30	0.22	3.75	0.06	0.13	0.89	0.05	0.13	0.01
	D	0.13	0.17	0.15	0.47	0.41	0.53	0.13	1.99	0.07	0.33	0.82	0.02	0.11	0.05
junctions	C	0.21	0.28	0.18	0.31	0.59	1.37	0.25	2.74	0.04	0.44	0.11	0.20	0.14	0.25
	D	0.18	0.18	0.15	0.26	0.23	0.83	0.22	1.29	0.02	0.02	0.06	0.04	0.07	0.07
end points	C	0.19	0.41	0.40	0.43	0.52	0.36	0.44	1.18	0.07	0.12	0.41	0.30	0.15	0.24
	D	0.19	0.21	0.75	0.21	0.22	0.75	0.10	0.60	0.04	0.17	0.29	0.06	0.19	0.05
segment lengths	C	0.07	0.13	0.04	0.18	0.14	0.34	0.08	0.60	0.00	0.01	0.00	0.02	0.09	0.02
	D	0.03	0.21	0.26	0.09	0.07	0.46	0.11	0.86	0.01	0.04	0.05	0.24	0.05	0.31

measurements		Branch Pruning ↓	Branch Pruning ↑	Branch Pruning o.
volume fraction	C	-	-	-
	D	-	-	-
network length	C	0.16	0.47	0.27
	D	0.05	0.35	0.08
segments	C	0.12	0.09	0.42
	D	0.10	0.15	0.32
skeletons	C	0.15	0.21	0.61
	D	0.20	0.55	0.57
junctions	C	0.08	0.06	0.31
	D	0.07	0.09	0.24
end points	C	1.22	0.92	4.22
	D	0.72	0.93	1.98
segment lengths	C	0.05	0.11	0.16
	D	0.15	0.00	0.23
